# Cholera

**Published:** 1856-01

**Authors:** Horatio N. Hollifield

**Affiliations:** Sandersville, Ga.


ARTICLE III.—*Cholera, by* HORATIO N. HOLLIFIELD, A. M., M. D., Sandersville, Ga.

It seems singular that the term "CHOLERA," which signifies an increased flow of bile, should be applied to a disease, one of the greatest peculiarities of which is the entire suppression of the biliary secretion.

My object in this communication is to make known the result of my inquiries, observations, and practical experience, to those who have not as yet had an opportunity of seeing, but who will, no doubt, at some future time, be called on to prescribe for this awful disease, the treatment of which requires promptness, assiduity, and skill.

The morbid phenomena which characterize cholera are of a formidable nature. The rapidity with which they run their course, and frequent fatality, makes it a subject of great interest to the members of the medical profession.

Having been engaged as resident physician in the Burlington County Hospital, of New Jersey, during the prevalence of the Cholera, I had a rare opportunity of studying it in its most fearful form. I took full notes of all the cases which came under my care and that of my colleagues, Doctors Coleman and Reed. While the epidemic continued I was, by duty, called on to act the nurse as well as the physician; not only to prescribe and direct, but also to assist; and often, after nights of toil, have I been rewarded by seeing my patients emerge from their dangerous state into one of comparative safety.

As a general rule, this disease is ushered in with a diarrhea, which often continues for several days, accompanied with loss of appetite, pain in the abdomen, failure of the digestive organs, general indisposition, pain in the head, languidness, and the alvine discharges; at first biliary, afterwards followed by those of a rice-water appearance. Sometimes the disease attacks without any warning or premonitory symptom, arrests the functions of life, and terminates fatally in a very short time. The characteristic symptoms of the disease, however, are as follows: Pain at the scrobiculus cordis, vomiting of a whitish fluid. The efforts at vomiting sometimes are ineffectual. The dejections watery; thirst very great; secretions suppressed, that of the urine entirely; absence of animal heat; the voice small and whispering; the skin, breath, and tongue cold; the eyes sunken; the integuments on the extremities wrinkled and of a bluish color; there is also great depression and despondency; spasms or cramps frequent; circulation in the heart and arteries greatly suppressed, and the alvine evacuations analagous to rice-water in look, and peculiar to this disease. The patient, if he recovers from the second stage, which is that of collapse or asphixia, is then often attacked with congestion or inflammation, caused by great vascular excitement, which sometimes follows the stage of collapse.

From this it will be seen we can divide the disease into three stages: 1st. That of incubation or irritation; 2d. That of asphixia or collapse; and 3d. That of reaction.

It is in the second stage only that the disease is to be positively recognized, and in the treatment of it we must always endeavor to follow the indications, to-wit: Overcome the irritability of the stomach and bowels; obtain healthy evacuations from the bladder and bowels; correct the state of the mucus coat of the stomach and intestines, and cause them to perfom their absorbent and secretory functions; excite the action of the heart and arteries; restore the animal heat, and overcome the spasmodic action of the muscles.

Of the 127 cases of this disease which came under my care last year, 102 recovered and 25 died; before I was employed in the institution, a number of cases occurred, of which the greater proportion proved fatal. The disease there assumed its most malignant form.

The following is a faithful description of a few of the cases, as they occurred in the hospital:

1st. John Morton, aged 42 years; been subject to fits for a long time; was attacked with diarrhea and vomiting on the 17th of September, at 9 o'clock, A. M. The abdomen was greatly distended and tympanitic; pulse 30 only perceptible in the carotids, where it was very perceptible; skin cold and clammy; breath cold. Administered stimulants; brandy, capsicum, and ammonia, and 1 pill every hour, of calomel, gr. v. and opii. gr. 1. At 11 o'clock lips blue, hands dark and shrivled, pulse fluttering; discharges from the rectum frequent; gave an enema of tinct. opii and gum water; applied sinapisms and frictions to the trunk and extremities. Two o'clock.—No pulse perceptible; extremities cold and stiff; respiration painful and laborious. The patient gradually sunk, and died at 9 o'clock in the evening, having been sick only twelve hours.—Post mortem examination: Great veinous conjestion in the upper and lower extremities; vessels of the brain very much engorged; the membranes thickened and inflamed, and the ventricles full of serum; lungs collapsed but not engorged; pancreas healthy; heart softer than common; the right ventricle full of blood of a dark color and very thick, resembling tar in appearance; stomach and intestines very red; the mucus coat softened and easily removed; the bladder was empty; spleen shriveled, and the muscles dry like jerked beef.

CASE 2.—Elizabeth Copperthawart, (a maniac,) aged 36; diarrhae and vomiting early in the morning, followed by cramps; pulse soft and slow; extremities cold; face flushed; tongue dry, and voice whispering; great tenderness at the scrobiculus cordis. Applied cataplasms to the abdomen and to the soles of the feet; administered a pill composed of 1 gr. of Opium, 5 gr. of calomel, 3 gr. of camphora, and 2 gr. of tannin each hour. At ten o'clock she seemed much better; hands blue and shriveled. Continued the pills every hour, in connexion with small doses of chloroform. The violent symptoms all abated by 8 o'clock the next day, when she was allowed a little gruel and brandy. She set up and considered herself well, although very weak; continued in this state for three days, when the vomiting and diarrhea returned, which was checked by means of small doses of chloroform, and an enema of opii and slack water. On the day following was much improved; the pulse good; vomiting and diarrhea entirely ceased. From this time she gradually improved, until she was entirely well.

3d. Jonathan Bishop, aged about 50 years; attacked on the 18th of September with pains and diarrhea; the eyes sunk and glassy; pulse at the wrist imperceptible; great pain in the epigastric region; bled him in the temporal artery, and administered opii tinc. lime catechu and chloroform, and applied mustard plasters to the extremities and abdomen. On the 19th much better; pulse perceptible—95; administered 5 gr. of calomel and of opii every three hours. On the 20th, so much improved as to be discharged.

4th. Matilda Lippencot, aged 35; complaining of swelling or fullness of the abdomen on the 17th of September, and pain at the scrobiculus cordis; on the 18th had a very violent diarrhea, and a feeling of sickness at the stomach; hands cold, blue, and shriveled; pulse very indistinct; gave small doses of chloroform every fifteen minutes, and one gr. of opii, v of calomel every hour, until she had taken 10 grs. of opii, and on the following day she was better; the hands had regained their natural color, warmth, and moisture; administered weak stimulants and allowed the patient very light diet; and in a few days she was considered well and able to resume her position as nurse.

Thus I have given a description of this disease, and a concise view of a few cases laboring under its baneful influence. The changes of weather during its continuance were very great, and the disease was, at all times, regarded by us as contagious.

